# Methyl 2-benzene­sulfonamido­benzoate

**DOI:** 10.1107/S1600536810025298

**Published:** 2010-07-07

**Authors:** Peter John, Onur Şahin, Islam Ullah Khan, Waqar Ahmad, Orhan Büyükgüngör

**Affiliations:** a Materials Chemistry Laboratry, Department of Chemistry, GC University, Lahore 54000, Pakistan; b Department of Physics, Ondokuz Mayıs University, TR-55139 Samsun, Turkey

## Abstract

In the title compound, C_14_H_13_NO_4_S, the conformation of the C—S—N—C segment is *gauche* and the two benzene rings are tilted relative to each other by 85.62 (8)°. An intra­molecular N—H⋯O hydrogen bond generates an *S*(6) ring and an C—H⋯O inter­action also occurs. In the crystal, inter­molecular C—H⋯O hydrogen bonds are observed, which link the mol­ecules into [100] *C*(7) chains.

## Related literature

For related structures, see: Khan *et al.* (2010 ▶); Sharif *et al.* (2010 ▶). For graph-set analysis, see: Bernstein *et al.* (1995 ▶).
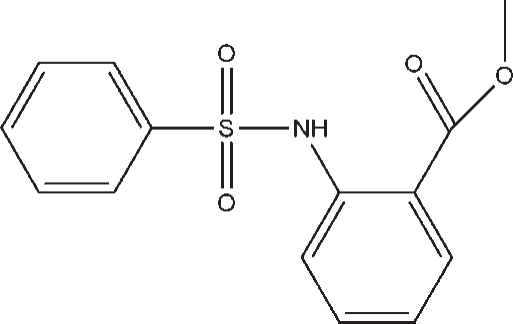


## Experimental

### 

#### Crystal data

C_14_H_13_NO_4_S
*M* *_r_* = 291.31
Triclinic, 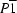

*a* = 8.341 (5) Å
*b* = 9.115 (3) Å
*c* = 10.000 (5) Å
α = 84.483 (5)°
β = 80.663 (5)°
γ = 66.674 (4)°
*V* = 688.5 (7) Å^3^
*Z* = 2
Mo *K*α radiation
μ = 0.25 mm^−1^
*T* = 296 K
0.18 × 0.10 × 0.07 mm

#### Data collection

Bruker APEXII CCD diffractometer
Absorption correction: multi-scan (*SADABS*; Bruker, 2007 ▶) *T*_min_ = 0.88, *T*_max_ = 0.99
12401 measured reflections
3387 independent reflections
2495 reflections with *I* > 2σ(*I*)
*R*_int_ = 0.030

#### Refinement

*R*[*F* ^2^ > 2σ(*F* ^2^)] = 0.043
*wR*(*F* ^2^) = 0.174
*S* = 1.15
3387 reflections
182 parameters
H-atom parameters constrained
Δρ_max_ = 0.59 e Å^−3^
Δρ_min_ = −0.59 e Å^−3^

### 

Data collection: *APEX2* (Bruker, 2007 ▶); cell refinement: *SAINT* (Bruker, 2007 ▶); data reduction: *SAINT*; program(s) used to solve structure: *SHELXS97* (Sheldrick, 2008 ▶); program(s) used to refine structure: *SHELXL97* (Sheldrick, 2008 ▶); molecular graphics: *ORTEP-3* (Farrugia, 1997 ▶); software used to prepare material for publication: *WinGX* (Farrugia, 1999 ▶).

## Supplementary Material

Crystal structure: contains datablocks global, I. DOI: 10.1107/S1600536810025298/hb5518sup1.cif

Structure factors: contains datablocks I. DOI: 10.1107/S1600536810025298/hb5518Isup2.hkl

Additional supplementary materials:  crystallographic information; 3D view; checkCIF report

## Figures and Tables

**Table 1:** Selected torsion angles (°)

C7—N1—S1—C1	−67.8 (2)

**Table 2:** Hydrogen-bond geometry (Å, °)

*D*—H⋯*A*	*D*—H	H⋯*A*	*D*⋯*A*	*D*—H⋯*A*
C8—H8⋯O2	0.93	2.45	3.062 (3)	124
N1—H1⋯O3	0.86	1.94	2.635 (3)	136
C8—H8⋯O2^i^	0.93	2.63	3.326 (3)	132
C4—H4⋯O1^ii^	0.93	2.48	3.265 (3)	142

Symmetry codes: (i) 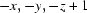; (ii) 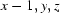.

**Fig. 1.**
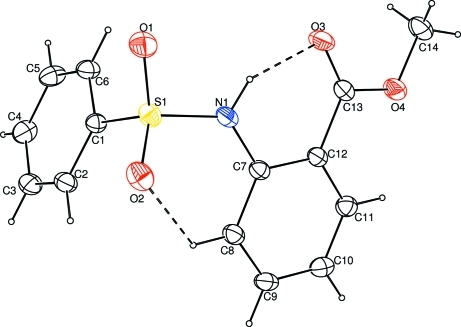
A view of the molecule of (I), showing displacement ellipsoids drawn at the 30% probability level.

**Fig. 2.**
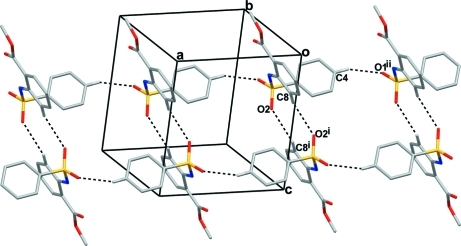
Part of the crystal structure of (I), showing the formation of R22(12) and R44(26) rings. H atoms not involved in these interactions have been omitted for clarity. (Symmetry codes as in Table 2).

## supplementary crystallographic information

### Comment

In continuation of our studies of sulfonamides synthesis (Khan *et al.*, 2010; Sharif *et al.*, 2010), herein, the crystal structure of title compound, (I), is described.

In the molecule of the title compound, (I), (Fig. 1), the coordination around the S atom is a distorted tetrahedral. The molecule is twisted at the S atom with the C1—SO_2_—NH—C7 torsion angle of -67.8 (2)°. In (I), the benzene ring C7—C12 is oriented with respect to the planar methyl ester moiety (O3/O4/C13/C14) and the benzene ring C1—C6 at dihedral angles of 5.29 (19)° and 85.62 (8)°, respectively. The dihedral angle between SO_2_ moiety and the benzene ring C1—C6 is 49.96 (12)°.

The intramolecular C8—H8···O2 and N1—H1···O3 hydrogen bonds produce S(6) rings (Bernstein *et al.*, 1995) (Fig. 1). The atom C8 in the molecule at (*x*, *y*, *z*) acts as a hydrogen-bond donor (Table 2) to atom O2^i^ so forming a centrosymmetric *R*_2_^2^(12) ring centred at (0, 0, 1/2). Atom C4 in the molecule at (*x*, *y*, *z*) acts as a hydrogen-bond donor to atom O1^ii^ so forming a C(7) chain running parallel to the [-100] direciton. The combination of the C—H···O hydrogen bonds along [100] generates a chain of edge-fused *R*_2_^2^(12) and *R*_4_^4^(26) rings.

### Experimental

To methyl anthranilate (428 µl, 3.3 mmol) in distilled water (10 ml) was added benzene sulfonyl chloride (421µl, 3.3 mmol) with stirring at room temperature while maintaining the pH of the reaction mixture at 8 using 3% sodium carbonate. The progress of the reaction was monitored by TLC. The precipitate formed in this way was washed with water, dried and crystallized from methanol to yield colourless blocks of (I).

### Refinement

All H atoms bound to C and N atoms were refined using a riding model, with C—H = 0.93Å and *U*_iso_(H) = 1.2*U*_eq_(C) for aromatic C atoms, C—H = 0.96Å and *U*_iso_(H) = 1.5*U*_eq_(C) for methyl C atom and N—H = 0.86Å and *U*_iso_(H) = 1.2*U*_eq_(C) for N atom.

### Crystal data

**Table:**

C_14_H_13_NO_4_S	*Z* = 2
*M**_r_* = 291.31	*F*(000) = 304
Triclinic, *P*1	*D*_x_ = 1.405 Mg m^−^^3^
Hall symbol: -P 1	Mo *K*α radiation, λ = 0.71073 Å
*a* = 8.341 (5) Å	Cell parameters from 4237 reflections
*b* = 9.115 (3) Å	θ = 2.4–26.0°
*c* = 10.000 (5) Å	µ = 0.25 mm^−^^1^
α = 84.483 (5)°	*T* = 296 K
β = 80.663 (5)°	Block, colourless
γ = 66.674 (4)°	0.18 × 0.10 × 0.07 mm
*V* = 688.5 (7) Å^3^	

### Data collection

**Table:**

Bruker APEXII CCD diffractometer	3387 independent reflections
Radiation source: fine-focus sealed tube	2495 reflections with *I* > 2σ(*I*)
graphite	*R*_int_ = 0.030
phi and ω scans	θ_max_ = 28.3°, θ_min_ = 2.1°
Absorption correction: multi-scan (*SADABS*; Bruker, 2007)	*h* = −11→10
*T*_min_ = 0.88, *T*_max_ = 0.99	*k* = −12→9
12401 measured reflections	*l* = −13→12

### Refinement

**Table:**

Refinement on *F*^2^	Primary atom site location: structure-invariant direct methods
Least-squares matrix: full	Secondary atom site location: difference Fourier map
*R*[*F*^2^ > 2σ(*F*^2^)] = 0.043	Hydrogen site location: inferred from neighbouring sites
*wR*(*F*^2^) = 0.174	H-atom parameters constrained
*S* = 1.15	*w* = 1/[σ^2^(*F*_o_^2^) + (0.1024*P*)^2^ + 0.0155*P*] where *P* = (*F*_o_^2^ + 2*F*_c_^2^)/3
3387 reflections	(Δ/σ)_max_ < 0.001
182 parameters	Δρ_max_ = 0.59 e Å^−^^3^
0 restraints	Δρ_min_ = −0.58 e Å^−^^3^

### Special details

**Table:**

Geometry. All e.s.d.'s (except the e.s.d. in the dihedral angle between two l.s. planes) are estimated using the full covariance matrix. The cell e.s.d.'s are taken into account individually in the estimation of e.s.d.'s in distances, angles and torsion angles; correlations between e.s.d.'s in cell parameters are only used when they are defined by crystal symmetry. An approximate (isotropic) treatment of cell e.s.d.'s is used for estimating e.s.d.'s involving l.s. planes.
Refinement. Refinement of *F*^2^ against ALL reflections. The weighted *R*-factor *wR* and goodness of fit *S* are based on *F*^2^, conventional *R*-factors *R* are based on *F*, with *F* set to zero for negative *F*^2^. The threshold expression of *F*^2^ > σ(*F*^2^) is used only for calculating *R*-factors(gt) *etc*. and is not relevant to the choice of reflections for refinement. *R*-factors based on *F*^2^ are statistically about twice as large as those based on *F*, and *R*- factors based on ALL data will be even larger.

### Fractional atomic coordinates and isotropic or equivalent isotropic displacement parameters (Å^2^)

**Table:**

	*x*	*y*	*z*	*U*_iso_*/*U*_eq_	
C1	−0.1640 (3)	0.3379 (2)	0.3275 (2)	0.0414 (4)	
C2	−0.2674 (3)	0.2666 (3)	0.4062 (2)	0.0515 (5)	
H2	−0.2178	0.1809	0.4653	0.062*	
C3	−0.4445 (3)	0.3239 (3)	0.3958 (3)	0.0590 (6)	
H3	−0.5150	0.2763	0.4479	0.071*	
C4	−0.5176 (3)	0.4511 (3)	0.3090 (3)	0.0616 (6)	
H4	−0.6373	0.4889	0.3023	0.074*	
C5	−0.4141 (3)	0.5228 (3)	0.2317 (3)	0.0613 (6)	
H5	−0.4642	0.6093	0.1735	0.074*	
C6	−0.2376 (3)	0.4666 (2)	0.2406 (2)	0.0510 (5)	
H6	−0.1675	0.5148	0.1886	0.061*	
C7	0.1571 (2)	0.0199 (2)	0.16147 (19)	0.0399 (4)	
C8	0.0860 (3)	−0.0706 (2)	0.2535 (2)	0.0477 (5)	
H8	0.0362	−0.0344	0.3403	0.057*	
C9	0.0893 (3)	−0.2135 (2)	0.2163 (2)	0.0510 (5)	
H9	0.0415	−0.2730	0.2785	0.061*	
C10	0.1620 (3)	−0.2690 (3)	0.0889 (3)	0.0550 (6)	
H10	0.1649	−0.3662	0.0652	0.066*	
C11	0.2308 (3)	−0.1804 (2)	−0.0039 (2)	0.0493 (5)	
H11	0.2787	−0.2179	−0.0906	0.059*	
C12	0.2295 (2)	−0.0359 (2)	0.0301 (2)	0.0404 (4)	
C13	0.2988 (3)	0.0601 (3)	−0.0732 (2)	0.0442 (5)	
C14	0.4176 (4)	0.0777 (3)	−0.3020 (2)	0.0681 (7)	
H20A	0.3188	0.1702	−0.3258	0.102*	
H20B	0.4699	0.0110	−0.3789	0.102*	
H20C	0.5032	0.1108	−0.2751	0.102*	
N1	0.1585 (2)	0.1651 (2)	0.19647 (18)	0.0490 (4)	
H1	0.2175	0.2063	0.1376	0.059*	
O1	0.1165 (2)	0.3981 (2)	0.31441 (17)	0.0633 (5)	
O2	0.1035 (2)	0.1602 (2)	0.44859 (15)	0.0600 (4)	
O3	0.3011 (2)	0.1886 (2)	−0.05568 (16)	0.0621 (5)	
O4	0.3592 (2)	−0.0106 (2)	−0.19141 (16)	0.0614 (4)	
S1	0.06389 (6)	0.26618 (6)	0.33289 (5)	0.0465 (2)	

### Atomic displacement parameters (Å^2^)

**Table:**

	*U*^11^	*U*^22^	*U*^33^	*U*^12^	*U*^13^	*U*^23^
C1	0.0432 (10)	0.0414 (9)	0.0435 (10)	−0.0214 (8)	−0.0027 (8)	−0.0023 (8)
C2	0.0492 (12)	0.0515 (11)	0.0524 (13)	−0.0221 (9)	−0.0011 (10)	0.0075 (10)
C3	0.0478 (12)	0.0580 (13)	0.0740 (16)	−0.0288 (10)	0.0046 (11)	−0.0002 (12)
C4	0.0431 (12)	0.0542 (12)	0.0885 (18)	−0.0185 (10)	−0.0122 (12)	−0.0020 (12)
C5	0.0570 (14)	0.0441 (11)	0.0837 (18)	−0.0190 (10)	−0.0209 (13)	0.0100 (11)
C6	0.0531 (12)	0.0470 (11)	0.0590 (13)	−0.0276 (9)	−0.0066 (10)	0.0050 (10)
C7	0.0346 (9)	0.0435 (9)	0.0400 (10)	−0.0146 (8)	−0.0072 (8)	0.0077 (8)
C8	0.0433 (11)	0.0507 (11)	0.0444 (11)	−0.0166 (9)	−0.0041 (8)	0.0096 (9)
C9	0.0491 (12)	0.0477 (11)	0.0574 (13)	−0.0231 (9)	−0.0103 (10)	0.0166 (9)
C10	0.0597 (14)	0.0450 (11)	0.0636 (14)	−0.0240 (10)	−0.0126 (11)	0.0056 (10)
C11	0.0524 (12)	0.0479 (11)	0.0469 (12)	−0.0188 (9)	−0.0077 (9)	0.0010 (9)
C12	0.0335 (9)	0.0450 (10)	0.0411 (11)	−0.0146 (8)	−0.0073 (8)	0.0073 (8)
C13	0.0383 (10)	0.0541 (11)	0.0403 (11)	−0.0198 (9)	−0.0042 (8)	0.0044 (9)
C14	0.0777 (17)	0.0807 (17)	0.0449 (14)	−0.0377 (14)	0.0081 (12)	0.0060 (12)
N1	0.0516 (10)	0.0531 (10)	0.0453 (10)	−0.0284 (8)	0.0055 (8)	0.0004 (8)
O1	0.0599 (10)	0.0711 (10)	0.0758 (12)	−0.0435 (8)	−0.0025 (8)	−0.0121 (9)
O2	0.0555 (9)	0.0795 (11)	0.0438 (9)	−0.0240 (8)	−0.0135 (7)	0.0058 (8)
O3	0.0829 (12)	0.0640 (10)	0.0500 (9)	−0.0465 (9)	0.0070 (8)	0.0011 (7)
O4	0.0775 (11)	0.0636 (9)	0.0425 (9)	−0.0333 (8)	0.0086 (7)	−0.0021 (7)
S1	0.0432 (3)	0.0566 (3)	0.0448 (3)	−0.0249 (2)	−0.0046 (2)	−0.0027 (2)

### Geometric parameters (Å, °)

**Table:**

C1—C2	1.382 (3)	C9—C10	1.371 (3)
C1—C6	1.383 (3)	C9—H9	0.9300
C1—S1	1.757 (2)	C10—C11	1.375 (3)
C2—C3	1.376 (3)	C10—H10	0.9300
C2—H2	0.9300	C11—C12	1.387 (3)
C3—C4	1.374 (3)	C11—H11	0.9300
C3—H3	0.9300	C12—C13	1.483 (3)
C4—C5	1.380 (3)	C13—O3	1.210 (3)
C4—H4	0.9300	C13—O4	1.327 (3)
C5—C6	1.369 (3)	C14—O4	1.440 (3)
C5—H5	0.9300	C14—H20A	0.9600
C6—H6	0.9300	C14—H20B	0.9600
C7—C8	1.395 (3)	C14—H20C	0.9600
C7—N1	1.406 (3)	N1—S1	1.6276 (18)
C7—C12	1.406 (3)	N1—H1	0.8600
C8—C9	1.377 (3)	O1—S1	1.4215 (17)
C8—H8	0.9300	O2—S1	1.4275 (17)
			
C2—C1—C6	120.60 (19)	C9—C10—H10	120.1
C2—C1—S1	121.10 (17)	C11—C10—H10	120.1
C6—C1—S1	118.29 (15)	C10—C11—C12	120.9 (2)
C3—C2—C1	119.1 (2)	C10—C11—H11	119.6
C3—C2—H2	120.4	C12—C11—H11	119.6
C1—C2—H2	120.4	C11—C12—C7	119.29 (18)
C4—C3—C2	120.4 (2)	C11—C12—C13	119.84 (19)
C4—C3—H3	119.8	C7—C12—C13	120.84 (18)
C2—C3—H3	119.8	O3—C13—O4	122.3 (2)
C3—C4—C5	120.2 (2)	O3—C13—C12	125.49 (19)
C3—C4—H4	119.9	O4—C13—C12	112.25 (18)
C5—C4—H4	119.9	O4—C14—H20A	109.5
C6—C5—C4	120.0 (2)	O4—C14—H20B	109.5
C6—C5—H5	120.0	H20A—C14—H20B	109.5
C4—C5—H5	120.0	O4—C14—H20C	109.5
C5—C6—C1	119.7 (2)	H20A—C14—H20C	109.5
C5—C6—H6	120.2	H20B—C14—H20C	109.5
C1—C6—H6	120.2	C7—N1—S1	129.02 (15)
C8—C7—N1	121.83 (18)	C7—N1—H1	115.5
C8—C7—C12	118.94 (19)	S1—N1—H1	115.5
N1—C7—C12	119.23 (17)	C13—O4—C14	116.87 (18)
C9—C8—C7	120.3 (2)	O1—S1—O2	120.11 (11)
C9—C8—H8	119.9	O1—S1—N1	103.89 (10)
C7—C8—H8	119.9	O2—S1—N1	108.85 (10)
C10—C9—C8	120.8 (2)	O1—S1—C1	108.53 (10)
C10—C9—H9	119.6	O2—S1—C1	108.40 (10)
C8—C9—H9	119.6	N1—S1—C1	106.20 (9)
C9—C10—C11	119.8 (2)		
			
C6—C1—C2—C3	0.8 (3)	N1—C7—C12—C13	−3.0 (3)
S1—C1—C2—C3	−178.17 (16)	C11—C12—C13—O3	178.5 (2)
C1—C2—C3—C4	−0.4 (3)	C7—C12—C13—O3	0.5 (3)
C2—C3—C4—C5	−0.2 (4)	C11—C12—C13—O4	−1.0 (3)
C3—C4—C5—C6	0.5 (4)	C7—C12—C13—O4	−179.01 (17)
C4—C5—C6—C1	0.0 (3)	C8—C7—N1—S1	−8.2 (3)
C2—C1—C6—C5	−0.6 (3)	C12—C7—N1—S1	171.85 (15)
S1—C1—C6—C5	178.41 (17)	O3—C13—O4—C14	−4.0 (3)
N1—C7—C8—C9	−179.05 (18)	C12—C13—O4—C14	175.53 (19)
C12—C7—C8—C9	0.9 (3)	C7—N1—S1—O1	177.77 (18)
C7—C8—C9—C10	0.0 (3)	C7—N1—S1—O2	48.7 (2)
C8—C9—C10—C11	−0.8 (3)	C7—N1—S1—C1	−67.8 (2)
C9—C10—C11—C12	0.8 (3)	C2—C1—S1—O1	−145.97 (17)
C10—C11—C12—C7	0.1 (3)	C6—C1—S1—O1	35.01 (19)
C10—C11—C12—C13	−177.87 (19)	C2—C1—S1—O2	−14.0 (2)
C8—C7—C12—C11	−0.9 (3)	C6—C1—S1—O2	167.02 (16)
N1—C7—C12—C11	179.00 (17)	C2—C1—S1—N1	102.86 (19)
C8—C7—C12—C13	177.03 (18)	C6—C1—S1—N1	−76.16 (18)

### Hydrogen-bond geometry (Å, °)

**Table:**

*D*—H···*A*	*D*—H	H···*A*	*D*···*A*	*D*—H···*A*
C8—H8···O2	0.93	2.45	3.062 (3)	124
N1—H1···O3	0.86	1.94	2.635 (3)	136
C8—H8···O2^i^	0.93	2.63	3.326 (3)	132
C4—H4···O1^ii^	0.93	2.48	3.265 (3)	142

Symmetry codes: (i) −*x*, −*y*, −*z*+1; (ii) *x*−1, *y*, *z*.
